# Intestinal Inflammation as a Dysbiosis of Energy Procurement: New Insights into an Old Topic

**DOI:** 10.1080/19490976.2021.1880241

**Published:** 2021-02-15

**Authors:** J. Scott Lee, Ruth X. Wang, Erica E. Alexeev, Sean P. Colgan

**Affiliations:** a Department of Medicine and the Mucosal Inflammation Program, University of Colorado School of Medicine, Aurora, United States; b Department of Gastroenterology, Inflammatory Bowel and Immunobiology Research Institute, Cedars-Sinai Medical Center, Los Angeles, United States

**Keywords:** Metabolism, microbiota, inflammation, creatine, butyrate, purine, colitis, epithelium

## Abstract

Inflammatory bowel disease (IBD) coincides with profound shifts in microbiota and host metabolic energy supply and demand. The gastrointestinal epithelium is anatomically positioned to provide a selective barrier between the anaerobic luminal microbiota and host lamina propria, with the microbiota and epithelium participating in an intricate energy exchange necessary for homeostasis. Maintenance and restoration of the barrier requires high energy flux and places significant demands on available substrates to generate ATP. It is recently appreciated that components of the microbiota contribute significantly to a multitude of biochemical pathways within and outside of the mucosa. Decades-old studies have appreciated that byproducts of the microbiota provide essential sources of energy to the intestinal epithelium, especially the colon. More recent work has unveiled the existence of numerous microbial-derived metabolites that support energy procurement within the mucosa. It is now appreciated that disease-associated shifts in the microbiota, termed dysbiosis, places significant demands on energy acquisition within the mucosa. Here, we review the topic of host- and microbial-derived components that influence tissue energetics in health and during disease.

## Introduction

The microbiota is defined as the constellation of microorganisms in coexistence with a given organism. Numerous studies over the last two decades have revealed these communities of microorganisms as essential for health. Found on both external and internal surfaces, including the skin, gastrointestinal tract, and oral mucosa, the microbiota is intimately integrated with the host and is as unique as our fingerprint. Although the human microbiota comprises of bacteria, fungi, archaea, and protozoa, bacteria vastly outnumber the other microorganisms by orders of magnitude, with the majority residing in the colon. A commonly referenced, initial estimation of the bacterial to human cell ratio in a body was 10:1, although the ratio has since been revisited and updated to ~1:1.^1^ This suggestion, that for every cell in a body that we consider as “us” there is a “them” in our gut, begets the age-old “how?” and “why?” questions, with the answers having great implication on how factors like lifestyle, diet, environment, and genetics shape the composition and activity of the gut microbiota, and ultimately our health and well-being.

Eukaryotes and microbes have long existed in mutually beneficial, symbiotic relationships with one another, relationships thought as fundamental to the development and evolution of multicellular life.^2^ For instance, an ancestor of modern eukaryotes engulfed an α-proteobacterium capable of oxidative phosphorylation, establishing an endosymbiotic relationship that led to the mitochondria as essential and defining organelles.^3^ The gut microbiota functions as another organ that resides in the host, albeit of microbial origin. This microbiota consists of organisms of different lineages that communicate with each other and the host, manipulates and redistributes energy, and mediates physiologically vital chemical transformations, providing us with essential functionalities upon which we depend.^4^ It has long been known that inflammatory bowel disease (IBD) is marked by a state of energy deficiency that involves dysbiosis of the composition and function of the gut microbiota concurrent with a loss of colonic epithelial barrier function.^5–7^ In this review, we will discuss newer and older literature regarding host- and microbial-derived components that constitute interdependent energy exchanges vital for colonic homeostasis and disease remission.

## The microbiota is compositionally and functionally dynamic

The human microbiota develops after birth and is shaped by a number of variables, including gestational age, delivery type, source of milk, weaning period, and exposure to antibiotics.^8^ Subsequently, the microbiota diversifies and stabilizes under the influence of environment, diet, genetics, and gut physiology into an adult-like composition by the age of three, growing to the highest cell density of any recorded ecosystem.^4^ Upon community stabilization, gut microbiota differences between individuals are shown to associate with many environmental and lifestyle aspects such as body mass index (BMI), exercise frequency, and, notably, diet.^8^ Gut microbes presumably have a tight co-evolutionary history with the host, while undergoing selective pressure from microbial competitors and the host alike.^9^ This environment is one that commonly produces a microbial community in which few groups occur in high abundance with several in low abundance.^10,11^ The vastly dominant gut microbial phyla are Firmicutes, Bacteroidetes, and Actinobacteria;^8,12^ and although the gut is home to great diversity at the species level, the microbiota is essentially comprised of divergent lineages of closely related bacteria from eight divisions.^4,11^

The composition of the gut microbiota is unique to each individual, much like a fingerprint. Numerous microbiome analyses over the years have provided useful insight into the functional capability of the microbiota. One such analysis revealed that the human distal gut microbiota is uniquely enriched in carbohydrate, amino acid, nucleotide, and coenzyme metabolic capability relative to other microbes. Further analyses identified that carbohydrate, nucleotide, amino acid, and energy metabolic pathways were among those enriched in the gut microbiome relative to the human genome, offering insight into the vast functional capability the microbiota can provide the host.^13^ By determining what substrates are available for fermentation, diet fundamentally influences microbiota composition based on differential microbial fermentative capabilities, with dietary-induced shifts in bacterial diversity and microbiota-derived end products observed as quickly as 24 h.^14^ For instance, it is well demonstrated that *Prevotella* is enriched in individuals with a plant-based diet rich in fiber and simple sugars, whereas *Bacteroides* dominates in those consuming a Western diet high in animal proteins and saturated fat.^15^ These dietary-linked microbiota compositions highlight the functional differences between *Prevotella* and *Bacteroides*, as *Prevotella* is more specialized for the degradation of plant material and relatively lacks lipolytic and proteolytic fermentative capabilities, whereas *Bacteroides* show more specialization for animal carbohydrates and proteolytic fermentative capacity.^16,17^ Symbiotic relationships such as that shared between the host and gut microbiota are dependent on the environment, with diet being a substantial environmental variable in this system that facilitates the expansion or restriction of certain microbial populations through selective competition for ingested nutrients.

Despite broad fermentative ability, the physiology of the gut microbiota is exceptionally suited for polysaccharide metabolism, reflecting the available resistant starch and non-starch polysaccharides (dietary fiber) gut microbes evolved around and are accustomed to harvesting for fuel in the gut microbiota-occupied niche. Humans produce ~17 gastrointestinal enzymes to mainly digest starch, whereas the gut microbiota produces thousands of complementary enzymes that depolymerize the xylan-, pectin-, and arabinose-containing carbohydrates remaining from consumed grains, fruits, vegetables, nuts, and legumes.^13,18^ In this, our microbiota offers us access to nutrients we otherwise could not. In addition to the resistant starch and non-starch polysaccharides mentioned above, a consistent carbohydrate source is provided by host epithelial goblet cells that secrete large amounts of highly glycosylated mucin proteins. The diverse capacity of gut microbes to utilize a wide variety of carbohydrates benefits the microbial community as a whole through cross-feeding, whereby the differential degradation abilities provide mono/oligosaccharides to the entire microbiota, affording a degree of flexibility in maintaining symbiotic function in a perpetually fluctuating nutrient environment.

Supplementing diet with non-digestible substrates, called prebiotics, for microbial fermentation have been reported to show beneficial effects in both of the IBDs ulcerative colitis (UC) and Crohn’s disease (CD), as well as chronic pouchitis.^19^ Current hypotheses on protective mechanisms of prebiotics include changes in the intestinal microbiota and improving the intestinal barrier.^19^ Several studies showed that prebiotics shift the intestinal microbiota toward a beneficial composition in both animal models and human studies, as they increase the quantity of beneficial bacteria, such as bifidobacteria and lactobacilli, at the loss of disease-inducing bacteria.^20,21^

## Microbial-derived metabolites and host health

Analogous studies of the microbiome at other levels corresponding to active genes (metatranscriptomes), proteins (metaproteomes), and metabolites (metabolomes) are comparatively lacking to microbiota composition studies, but may be more relevant in understanding how the gut microbiota defines and contributes to host function.^22^ A beneficially functional gut microbiota requires continuous adaptation to the variable nature of food intake, in that the microbiota is capable of processing a range of molecules into the compounds required by the host. As discussed above, this capability is achieved in part through compositional plasticity and cross-feeding, but also through a high level of functional redundancy.^23,24^ This functional redundancy is in part due to horizontal gene transfer occurring 25-times more often in gut bacteria than between other bacteria, stimulating the evolution and selection of specific core functions in the intestinal ecosystem.^13,22,25^ Indeed, one of the most prominent findings of the Human Microbiome Project was that although taxonomic composition significantly differs among individuals, the abundances of metabolic pathways are relatively consistent.^26,27^ In this, two microbiotas differing in composition at the species and/or genetic level may produce similar protein and metabolite profiles.^22,24^ Over the years, attention has shifted from microbiome-based, associative studies toward the mechanistic determination of the molecular interactions between the microbiota and host. An important insight resulting from these studies is that many microbiota–host interactions are mediated by metabolites secreted or modified by the microbiota and/or host.^28^ A recent study comparing germ-free and colonized mice revealed that such microbial metabolites affect the chemistry of all organ systems, highlighting how the gut microbiota signals to distant organs and influences whole-body metabolism.^29^

The importance of intestinal homeostasis to whole-body health is manifested by numerous gut-organ axes, where disease in other organs often occurs with gastrointestinal (GI) diseases. It is appreciated that dysbiosis of the gut microbiota is not limited to IBD and associates with irritable bowel syndrome (IBS), allergies, asthma, metabolic syndrome, and cardiovascular disease.^30–33^ Commonly, these GI diseases present a multifaceted pathophysiology involving dysbiosis of the composition and metabolism of the microbiota and intestinal epithelial barrier loss, with this increased epithelial permeability allowing inappropriate immunological stimulation by luminal antigens. In the chronic disease state of UC, these factors perpetuate each other in a convoluted relationship between the dysbiosis-induced loss of microbiota-derived metabolites, dysfunctional epithelial barrier, and unresolving inflammation.^34^ This intricate relationship motivates the search for therapeutic approaches that address both host processes and the microbiota.^35^ As epithelial barrier is responsible for creating a habitat that promotes a healthy microbiota, isolating that microbiota from the host immune system, and coordinating crosstalk between the two,^36,37^ targeting epithelial barrier restoration may have such a pleiotropic response. To do so necessitates defining the contribution of microbiota-derived metabolites to gut barrier function and the dysbiosis-induced shifts of those metabolites in disease.

## The high energy demand of mucosal barrier function

The GI tract is tasked with managing nutrient and waste flux in a manner that protects the host from luminal pathogenic microbes and antigenic materials. Intestinal homeostasis requires substantial energy input, with the gut devoted ~20% of total cardiac output while consuming 10–20% of the available oxygen.^38–40^ The microbiota is also a substantial contributor to host energy procurement. This is exemplified in that germ-free (GF) mice lacking a microbiota are lean in comparison to conventionally-raised (CR) mice, with the colonization of GF mice inducing rapid weight gain and increased adiposity.^41^ The contribution of the microbiota to the human energy requirement is estimated to be 5–10%,^42^ with a considerable local impact on the large intestine. For example, analyses of the total available energy (TAE), a metric that accounts for the total available chemical energy in a system as ADP, ATP, and phosphocreatine,^43^ from colon tissue extracts reveal that GF mice have ~55% (*p* < .001) of the TAE shown in CR counterparts.^44^ Much of this microbiota-derived energy is devoted to a monolayer of intestinal epithelial cells (IECs) residing at the frontier of the microbiota–host interface that construct and maintain a mutually beneficial, selective barrier between the gut microbiota and host. In doing so, these IECs establish the first line of defense against pathogen infection, but also provide fuel and habitat for microbial symbionts. Two primary differentiated host epithelial cell types are responsible for constructing and maintaining barrier in the large intestine – the goblet cell and enterocyte – with distinct and essential contributions to this key component of gut homeostasis.

### Mucus barrier

Goblet cells secrete large amounts of mucin proteins to construct a barrier that physically separates the epithelium and microbiota. A healthy colonic mucus barrier has two layers – a dense, stratified inner layer impenetrable to most luminal microbes and an outer, less dense layer containing microbes and dietary material.^45^ In a healthy colon, mucins are continuously secreted at a rate of ~2 – 4 μm/min, creating a flow that physically repels microbes and turns over the mucus barrier hourly.^46^ The transition from the inner to the outer layer is in part endogenously controlled to assist mucin clearance, and allow mucin to provide lubrication for the fecal stream.^47^ The prominent secreted mucin is MUC2, which has a high level of O-glycosylation that accounts for ~80% of the total molecular weight of intestinal mucus.^48^ This glycosylation helps shield the mucin protein backbone from host and microbe proteases and binds the water necessary for gel formation. The integrity of the mucus layer is critical for health, as it is the first intestinal structure that a pathogen must overcome to establish infection. Genetic ablation of MUC2 expression or loss of O-glycans in mice incites spontaneous colitis and sometimes cancer.^49,50^ Correspondingly, structural weakening of the mucus barrier is implicated as an early event in UC pathogenesis,^51^ while patients with active UC have an abnormally penetrable inner mucus layer and altered O-glycosylation profile.^52,53^

The secretion of large amounts of mucin protein is a substantial energy and nucleotide demanding process at multiple levels. Fundamentally, nucleotide templates are required for ribosomal RNA generation and messenger RNA transcription, with the need for nucleotides most prominent during transcription.^54^ After which, the translation of secretory proteins on the endoplasmic reticulum (ER) is fueled by GTP hydrolysis, followed by numerous ATP consuming processes like protein translocation, folding, post-translational modifications, and trafficking.^55^ The extensive nucleotide demanding process of O-glycosylation occurs once the mucin reaches the Golgi, requiring nucleotide sugar precursors to sequentially add single monosaccharides.^56^ Given the high energy requirement for ER function in secretory cells, energy deprivation disrupts protein folding and glycosylation, causing ER stress. To overcome this imbalance in protein folding capacity, ER stress activates a pathway called the unfolded protein response (UPR) that engages transcription factors and enzymes in an effort to reestablish ER homeostasis.^57^ Notably, several proteins that sense and respond to ER stress are O-glycosylated, which is thought to function to relay the status of the Golgi and secretory apparatus to regulate the UPR.^58^ The dependency of the ER on energy balance is underscored by exquisite sensitivity and coordination of responses to energy fluctuations. The UPR transcriptionally regulates glucose synthesis and breakdown genes, in addition to having important roles in fatty acid and cholesterol metabolism.^59^ Furthermore, the ER directly communicates with mitochondria to increase ATP regeneration during times of energetic demand.^60^ Prolonged ER stress induces inflammation and apoptosis, as demonstrated by mice aberrant in MUC2 oligomerization leading to MUC2 precursor accumulation-induced ER stress, increased apoptosis, and spontaneous colitis.^61^ The colitis developed in these mice exhibited the decreased goblet cell number, decreased MUC2 production and secretion, MUC2 precursor accumulation, and increased ER stress phenotypes characteristic of UC.^62^ Interestingly, colonic epithelial defects in the key UPR component X-box binding protein 1 (*XBP1*) are reported in UC, identifying the ER stress pathway as a common genetic contributor to the disease.^63^ Given the intimate relationship with and dependence on energy metabolism for ER function and the state of energy deficiency of the colonic mucosa during UC,^5–7^ it is reasonable that poor energy balance may be a significant contributor to the penetrability of the mucus barrier during disease that must be addressed for healing.

### Apical junction complex

Colonic enterocytes contribute to barrier function by forming intercellular adhesion complexes mediated by tight and adherens junctions, termed the apical junction complex (AJC). The most apical tight junctions (TJs) are the primary regulators of paracellular permeability to solutes and macromolecules (gate function), while polarizing the enterocytes into apical and basolateral regions that establish a gradient between the intestinal lumen and basolateral membrane (fence function).^64,65^ Adherens junctions (AJs) provide the strong adhesive bonds responsible for maintaining cellular proximity and junctional complex stability.^66^ Loss of AJs disrupts cellular contacts, polarization, and differentiation, while inducing premature apoptosis.^66,67^ The AJs are made up of a family of transmembrane proteins called cadherins that form strong, homotypic interactions with molecules from neighboring cells. The cytoplasmic end of cadherins interacts with catenin proteins that link the AJ to the cellular cytoskeleton and a perijunctional actomyosin ring. This AJ system provides the stability necessary for TJ formation and resultant sealing of the paracellular space.

Small molecules, including potential antigens, easily diffuse through the mucus layers. Epithelial paracellular flow is typically more permeable than transcellular flux, conferring TJs as the rate limiters of transepithelial transport and principle determinant of mucosal permeability.^66^ TJs are multi-protein complexes, with the most important members from the claudin family. Peripheral membrane or scaffolding proteins such as the zona occludins (ZO) are crucial to TJ formation, notably through linking TJ proteins to the actin cytoskeleton. At least two independently regulated means of transport across TJs are identified. The leak pathway allows passage of large solutes, even molecules as large as proteins and proinflammatory bacterial lipopolysaccharides.^68,69^ Flux across this pathway is increased by proinflammatory cytokines such as interferon-γ and tumor necrosis factor, which are highly expressed in the chronically inflamed intestine.^70,71^ The other pathway represents the canonical association with TJ function, in which small pores formed by claudin proteins exclude molecules larger than 4 angstroms in a charge selective manner.^68,69^ Both size and charge selection can be independently or jointly regulated in response to various physiological or pathophysiological stimuli.^66^ Dysregulation of the TJ and thus paracellular flux is another hallmark of UC.^71–73^ For instance, a study that measured epithelial resistance as a metric of TJ function revealed an 80% reduction in resistance in samples from UC patients with an inflamed colon.^74^ Perhaps unsurprisingly, restoration of homeostatic TJ characteristics correlates with quiescent UC and mucosal healing, notably in part through increased expression of the actin associating ZO proteins.^75^

Studies using ATP depletion models suggest that the AJC commands substantial energy to control paracellular flux.^76^ Much of this energy is devoted to the cytoskeletal components tasked with junctional regulation, which connect to a network that transduces adhesive and mechanical signals from the membrane, into the cell, and back to mediate AJC regulation.^77,78^ Accommodating the various cell morphologies and movements that occur in a monolayer requires junctions to be both strong and plastic, a functionality that requires an exceptionally active cytoskeleton rich in actin filaments working to stabilize and cycle junction proteins.^64,77,79^ To this end, the AJC complex is supported by a network of TJ-associating F-actin bundles and the dense circumferential actomyosin ring contiguous with AJs that forms one of the most organized and active actin networks found in nature.^80^ This ATP-dependent actomyosin ring provides stability and intercellular tension that forces paracellular flux through the TJ,^73,80,81^ while ATP-fueled turnover, or treadmilling, of the F-actin bundles facilitates the extension and contraction of actin filaments to, in part, cycle TJ proteins.^82–85^ Not only does mucosal healing in UC necessitate TJ reformation and homeostatic regulation, but also epithelial migration for wound closure and cellular polarization. It was demonstrated that T84 model IECs devote nearly 20% of cellular TAE to cytoskeletal activity in such a wound healing scenario.^43^ Given that these processes require exceptional cytoskeletal capacity and energy supply to drive restoration and maintenance of the AJC, they are likely hindered by the energy-deficient state associated with UC.^5–7^

## Interdependent energy circuits of the microbiota and host epithelium

All cells require a source of energy to maintain cellular functions, growth, and reproduction. Fundamentally, the microbiota bioreactor is a system that transforms otherwise-indigestible complex carbohydrates into energy that fuels bacterial communities and the host, producing a remarkable number of metabolites in the process. It is of ongoing interest to define what metabolites are microbial-derived and the responsible taxa.^27^ Microbes in the gut community compete for nutrients, but also participate in complex cross-feeding relationships in which the excreted product from one strain is the preferred energy source for another.^86^ Deficiency in one component of this intricate cross-feeding relationship can have extended consequence across the bacterial community, inducing global shifts in microbiota composition and metabolism. Such a deficiency may then deprive the colonic epithelium of metabolites upon which it relies for energy procurement and barrier function. Restoring homeostasis to this multifaceted, interdependent system calls for an equal therapeutic approach. Given that maintenance of energetic relationships is paramount to microbiota and host function in health, understanding and identifying breaks in these metabolite-mediated energy circuits presents a means to address disease.

Humans consume a wide range of complex carbohydrates, many of these dietary polysaccharides endure digestion and pass through the stomach and small intestine. These resistant starches (RS) and non-starch polysaccharides (NSP, the major component of dietary fiber) reach the colon, providing the primary energy source for the system, where they are fermented by the microbiota to short-chain fatty acids (SCFAs). Even though NSP completely resists digestion by intrinsic human intestinal digestive enzymes, its intake may account for only 25% of the calculated production of SCFAs.^87^ This deficit is partially filled by oligosaccharides, but dietary RS are often the single largest contributor to colonic microbial growth.^88^ These RS are fermented by specialized microorganisms to ultimately produce the SCFAs acetate, propionate, and butyrate. Succinate and lactate are also prominent fermentation products but generally do not accumulate higher than 5 mM in healthy adults, as they are substrates for other bacteria, including propionate and butyrate producers.^89–91^ Protein fermentation can also occur in the distal colon, as various amino acids can be used to produce SCFAs, but is less favored to carbohydrate substrate and also generates potentially toxic metabolites such as ammonia, indoles, and phenols.^89^ Such an imbalance between SCFAs and toxic metabolites may contribute to the pathogenesis of IBD and colon cancer.^92^

Colonic SCFAs exist in molar ratios of approximately 60:20:20 for acetate:propionate:butyrate, with total SCFAs reaching up to 140 mM in the proximal colon.^87^ Acetate is a common fermentation product of many gut anaerobes and is also produced by reductive acetogenesis, accounting for the greater accumulation, while propionate and butyrate are produced by distinct bacterial subsets.^89^ These SCFAs are efficiently taken up by the gut mucosa, with only 5–10% estimated to be excreted in feces, and have significant impact on host physiology as energy substrates, regulators of gene expression, and signaling molecules recognized by specific receptors.^89,93–95^ However, these SCFAs are differential in their influences, fate, and tissue distribution. For instance, propionate contributes to gluconeogenesis in the liver while acetate reaches highest concentrations in the blood and primarily transports to muscle.^93,96^ This review will center around butyrate production as this SCFA is the most increased by RS consumption,^97^ the preferential fuel source of the colonic mucosa, and a foundational contributor to colonic microbiota–host energy circuits.

### Microbiota-derived butyrate

Clostridia are the major butyrate-producing class, and particularly, *Eubacterium rectale, Eubacterium hallii*, and *Faecalibacterium prausnitzii* as among some of the most abundant and dominant butyrate-producing species, and encompass the primary biosynthetic routes utilized for butyrate production.^89,96,98^ Notably, *E. rectale* and *F. prausnitzii* are capable of producing butyrate from the oligosaccharide inulin in an acetate-dependent manner, yet both appear limited in their capacity or are unable to degrade RS.^89,98–100^ It is appreciated that the primary degradation of RS is conducted by two currently known species – *Ruminococcus bromii* and *Bifidobacterium adolescentis* (and other related *Bifidobacterium* species).^97^ As such, these primary degraders are responsible for producing the available mono/oligosaccharides from RS and acetate needed by *E. rectale* and *F. prausnitzii* for butyrate production, and therefore are regarded as “keystone species.”^99,101^ Accordingly, increases in the abundance of *R. bromii* by diets supplemented with RS are concomitant with increases in *E. rectale* and butyrate.^99^ The human bacterium *Ruminococcus champanellensis* is a member of this very important keystone group due to an ability to degrade the NSP cellulose.^88^ Butyrate production from *E. hallii* is distinguished in that the bacterium utilizes lactate and acetate as substrates.^91,98^ Lactate is a common end product of bacterial fermentation produced, among others, by the genera *Lactobacillus* and *Bifidobacterium*, which are regarded as key members of the gut microbiota due to their health-promoting capabilities.^98^ The keystone species *B. adolescentis* degrades RS to produce lactate in addition to acetate through a unique metabolic pathway to *Bifidobacterium* named the “bifid shunt,” functioning as a primary degrader for butyrate production by *E. hallii.*

As mentioned earlier, colonic goblet cells secrete large amounts of highly glycosylated mucin proteins that provide a significant carbohydrate source for the microbiota. Only *Bacteroides thetaiotaomicron, Ruminococcus gnavus, Ruminococcus torques, Bifidobacterium bifidum*, and *Akkermansia muciniphilia* are known as capable of partial or full mucin degradation.^102^ With glycans constituting 80% of the dry weight of mucin and the microbiota able to access this fuel source, it is as though the colonic epithelium itself produces a prebiotic. Since discovery as an abundant member of the microbiota in 2004, *A. muciniphilia* has gained attention for its therapeutic potential in treating UC.^103^ The mucin protein backbone has primarily O-glycosylated and some N-glycosylated chains of 2 to 12 monosaccharides of mostly galactose, fucose, N-acetylgalactosamine, N-acetylglucosamine, mannose, and sialic acid.^102^ Mucin degradation by *A. muciniphilia* results in the release of oligosaccharides and production of acetate and propionate fueled by an impressive ability to utilize up to 85% of the complex mucin structure as the sole carbon and nitrogen source.^102^ Because of this capacity to access mucin glycans and cross-feed the microbial community, *A. muciniphilia* is thought of as a keystone species. Support for this is found through the concomitant enrichment of mucolytic and non-mucolytic butyrogenic bacteria in the mucus environment and was demonstrated by *A. muciniphilia* supporting the butyrate-producing gut commensal *Anaerostipes caccae*.^104^ Furthermore, *Clostridium* cluster XIVa species, encompassing well-known butyrate producers such as *E. rectale* and *E. hallii*, account for nearly 60% of the mucin-adhered microbiota, conferring *A. muciniphilia* as a local primary degrader with this proximity to the colonic epithelium thought to facilitate butyrate bioavailability.^105^

Butyrate has a wide range of influence over cellular processes in the colonic mucosa that include G-protein coupled receptor signaling and histone deacetylase (HDAC) inhibition to regulate gene expression, both of which mitigate chronic inflammatory responses.^96,106^ To maintain focus on microbiota–host energy circuits, the scope of this review will be primarily limited to the role of butyrate in epithelial energy and barrier function.

Butyrate is the preferential fuel source of the colonic epithelium, with oxidation of this SCFA accounting for over 70% of the cellular oxygen consumption in the distal colon.^107^ Because most colonic butyrate exists in the dissociated form, apical epithelial transport from the lumen and into the cell is facilitated via several transporters, mainly the SCFA-HCO_3_^−^ exchange, monocarboxylate transporter isoform 1 (MCT1), and sodium-coupled monocarboxylate (SMCT1) transporters. A steep concentration gradient exists between luminal and systemic butyrate concentrations, with systemic availability of colonic-administered butyrate shown to be 2%.^108^ This is in part due to different affinities of colonocyte apical and basolateral SCFA-HCO_3_^−^ exchange transporters for butyrate (K_m_ = 1.5 mM apically and 17.5 mM basolaterally), through which butyrate is sequestered for energy procurement.^108–110^ As an energy substrate, butyrate undergoes β-oxidation to form acetyl-CoA, which enters into the TCA cycle to produce the NADH that drives the electron transport chain (ETC) and oxygen consumption to ultimately regenerate ATP. This energetic supply provides critical support to the cytoskeleton and thus barrier, bestowing IECs an unsurpassed capacity to rapidly polarize and form strong AJCs.^111^ A substantial contribution of butyrate to barrier function stems from ATP provision to the cytoskeleton, but also through upregulating the expression of actin-binding proteins like synaptopodin by HDAC inhibition and activation of other transcription factors like STAT3, SP1, and AMPK that induce genes encoding for TJ components.^106,111,112^

**Figure 1.**
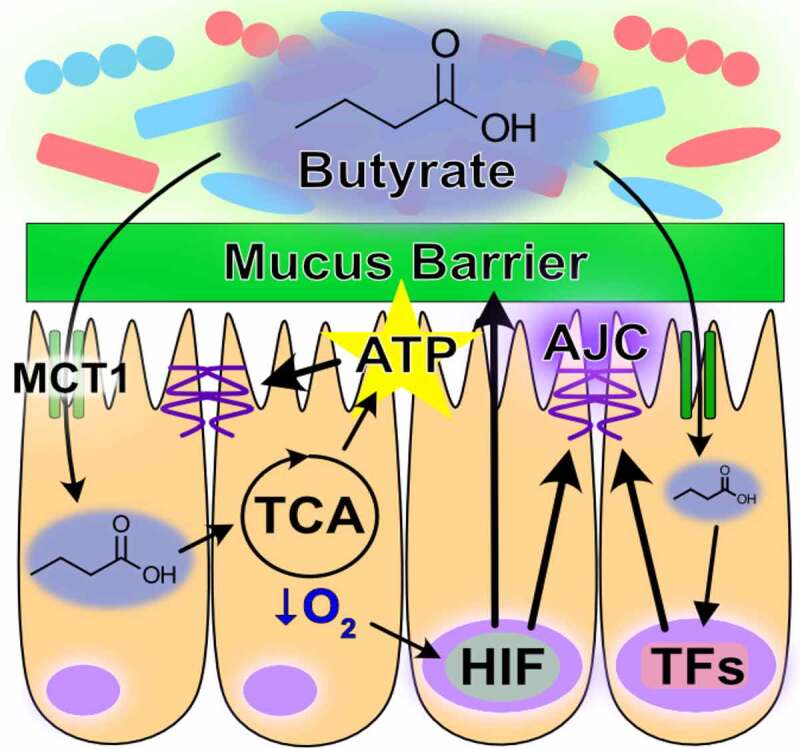
*Role of butyrate in intestinal homeostasis*. Microbial-derived butyrate is a fuel source for intestinal epithelial cells and promotes barrier through gene regulation (MCT1, monocarboxylate transporter isoform 1; TCA, tricarboxylic acid; AJC, apical junction complex; ATP, adenosine triphosphate; HIF, hypoxia-inducible factor; TFs, transcription factors)

The oxygen consumed for butyrate metabolism is an important determinant of intestinal homeostasis. In this, not only does microbiota-derived butyrate serve as the primary fuel source for the colon, but epithelial butyrate metabolism also shapes the gut milieu. The colonic mucosa exists in a state of physiologic hypoxia, in part due to anoxic colonic lumen but also from the oxygen consumption resulting from butyrate metabolism.^113,114^ This oxygen depletion stabilizes hypoxia-inducible factor (HIF), a transcription factor that regulates many genes important for intestinal barrier function, such as the TJ protein claudin 1 (CLDN1).^115^ Oxygen concentrations in colon tissue range from ~1% near the lumen and increase to ~5-10% in the vascularized submucosa and muscle layers.^113^ The ETC can function at near anoxia and is not limited by intracellular oxygen until levels reach 0.3%.^116,117^ Instead, flux through the ETC during hypoxia is attenuated by HIF-dependent and HIF-independent mechanisms, a major benefit being decreased formation of mitochondrial reactive oxygen species that can incur cellular injury.^118–121^ HIF modulates glucose metabolism by increasing glucose uptake and flux through glycolysis, while shunting the resulting pyruvate from conversion into acetyl-CoA and entrance into the TCA cycle toward lactate production and efflux.^118,122–128^ In doing so, butyrate-induced HIF stabilization molds metabolism to confer butyrate as the primary source of acetyl-CoA for the TCA cycle and thus mitochondrial ATP regeneration in the healthy colon. Additionally, the HIF-mediated upregulation of glycolysis also drives ATP regeneration, further promoting epithelial energy balance and function. The energy provided from butyrate oxidation also supports mucin production complemented by the upregulation of MUC2 expression by HIF, providing fuel and habitat for the microbiota and altogether contributing to mucus and AJC barriers (Table 1).^127,129^ As a whole, microbiota-derived butyrate is a critical component and modulator of epithelial energy metabolism that fundamentally molds and contributes to a homeostatic colonic environment (Figure 1).

**Table 1.** Origins and influences of butyrate, hypoxanthine, and creatine on gut energy metabolism and barrier function

Metabolite	Primary Source	Energetic Role	Barrier Contribution
Butyrate	*Eubacterium rectale* *Eubacterium hallii* *Faecalibacterium prausnitzii* *Roseburia inulinivorans* *Roseburia intestinalis* *Anaerostipes hadrus* *Coprococcus eutactus* *Coprococcus catus* *Subdoligranulum variabile^89^*	β-oxidation of fatty acid for mitochondrial-driven ATP regeneration.^107^ Mitochondrial oxygen consumption-induced HIF stabilization increases glycolysis-driven ATP regeneration.^118,122–128^	ATP regeneration^107^ Induction of cytoskeletal binding proteins^111^ Induction of TJ proteins^106,112,115^ Induction of MUC2^128,129^ Induction of creatine kinases^132^
Hypoxanthine	Associates with *Barnesiella* and *Prevotella*,^130^ TBD*	ATP and GTP generation.^43,44^ Microbial substrate,^131^ TBD*	Cytoskeletal ATP^43^ AJC formation and stability^43^ Mucin generation and mucus barrier sterile integrity^44^
Creatine	Diet and Endogenous Biosynthesis	Temporal and spatial ATP buffer. Microbial carbon and nitrogen source.^133^	ATP buffering/regeneration^132,134,135^ AJC formation and stability^132,134,135^

TBD*, to be determined

### Microbiota-sourced purines

The purine nucleobase hypoxanthine is a significant product of the gut microbiota that provides a readily available substrate for efficient purine nucleotide biogenesis.^44,130^ Through experiments in which mice were treated with gentamicin, ceftriaxone, and a combination of the two followed by fecal microbiome and metabolomic analyses, fecal hypoxanthine abundance most strongly associated with the genus *Barnesiella*, and to a lesser extent *Prevotella*, identifying potential sources of hypoxanthine production by the microbiota.^130^ The rapid cellular turnover and need to secrete large amounts of highly glycosylated mucins characteristic of the colonic epithelium is especially nucleotide demanding. Substantial nucleotide template is required to duplicate the genome (DNA) for proliferation and to support the ribosomal RNA generation, messenger RNA transcription, and glycosylation required for sustained mucin secretion.^54,56^ This purine nucleotide requisite is met via two pathways – the salvage pathway, which is true to its namesake and utilizes exogenous purine as substrate for nucleotide generation, and the ATP and nutrient-consuming *de novo* pathway which sequentially constructs purine nucleotides from phosphoribosyl pyrophosphate (PRPP), at the very costly expense of 5 ATP per purine molecule produced. Previous studies have demonstrated that the gut mucosa preferentially salvages purines in lieu of the energy and nutrient-consuming *de novo* pathway in the presence of available purine substrate.^44^ This salvage is an efficient, resource-conserving alternative to *de novo* purine biosynthesis.

**Figure 2.**
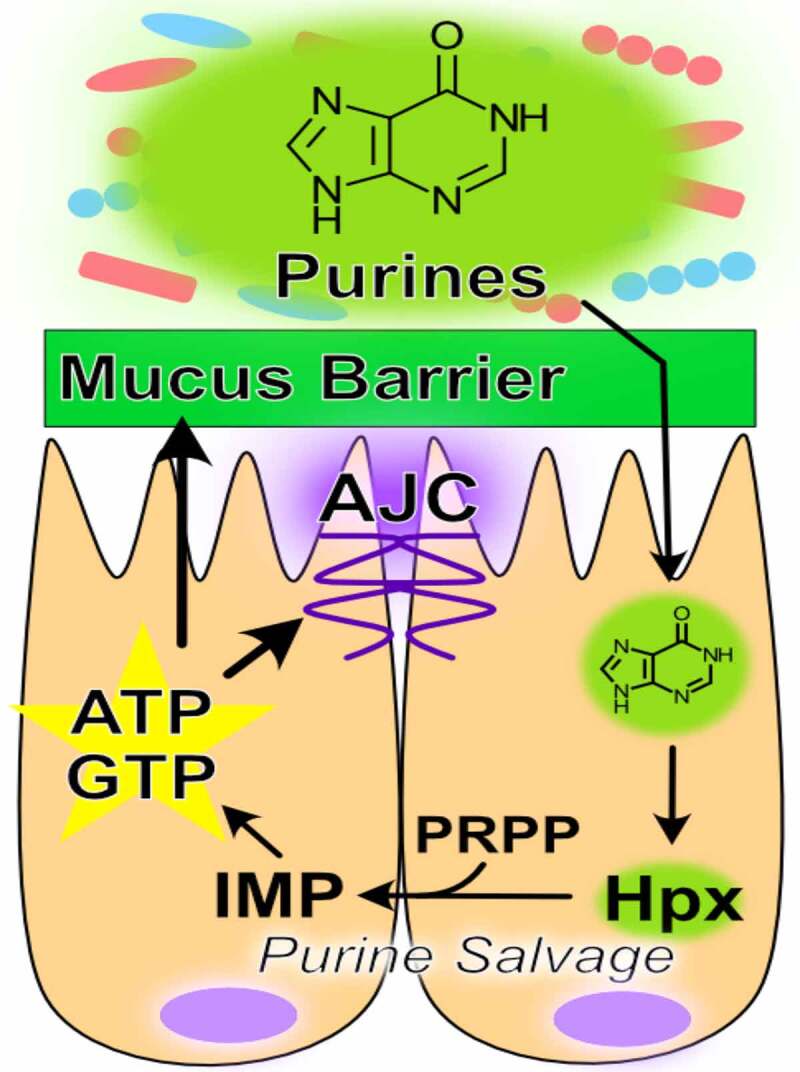
*Purine metabolism promotes barrier function*. Hypoxanthine from the microbiota is salvaged for energy and nucleotide biosynthesis in the colon. This energy and nucleotide source fuels cytoskeletal support of the apical junction complex and drives mucin generation (AJC, apical junction complex; IMP, inosine monophosphate; Hpx, hypoxanthine; PRPP, phosphoribosyl pyrophosphate; ATP, adenosine triphosphate; GTP, guanosine triphosphate)

Hypoxanthine is readily salvaged by IECs to support energy balance and nucleotide biosynthesis.^43,44^ For example, colonic enterocyte model T84 cells show a substantial decrease in AJC barrier resistance when subjected to hypoxia, an energetically depleting state of oxygen deprivation representative of their natural environment. Hypoxanthine supplementation significantly increased the total available energy (TAE) in hypoxic cells, concomitant with complete recovery of AJC function. Further analyses revealed that the energetic benefit afforded by hypoxanthine supplementation promoted actin polymerization and AJC stability, and increased AJC formation from a depolarized cell state.^43^ Extended *in vivo* studies revealed that depletion of microbial purine production through streptomycin treatment significantly decreased colonic tissue purine levels, and that those purine levels could be restored by colonization with streptomycin-resistant, purine-producing bacteria.^44^ The colonic mucosa was found to be dependent upon this microbiota-sourced purine for energy balance and nucleotide biogenesis during dextran sodium sulfate (DSS)-induced colitis. Colonic tissue depleted of microbiota-sourced purines showed increased ER stress concurrent with loss of inner mucus layer thickness and sterile integrity during the colitic insult, rendering the barrier more penetrable to microbes. Tissue energy balance and the sterile integrity of the mucus barrier was recovered, and ER stress alleviated, by reconstitution of exogenous purine supply through colonization with purine-producing bacterial or oral hypoxanthine supplementation, identifying purines as a limiting substrate in such processes.^44^ In analogous work, lower amounts of hypoxanthine were recently observed in the stools of IBS patients.^131^ Notably, the microbiota was found to also use hypoxanthine as a substrate, endowing the metabolite as a cross-feeding substrate. Additionally, IBS patients appeared to have decreased fecal hypoxanthine as a result in part of increased microbial utilization and breakdown, particularly by *Lachnospiraceae*, and the ensuing purine starvation in the colonic epithelium identified as a potential new mechanism underlying IBS.^131^ Overall, microbiota-sourced purines appear as substantial contributors to and critical substrates for colonic energy homeostasis, barrier function, and potentially a healthy microbiota, warranting further characterization (Table 1, Figure 2).

### Microbiota and host creatine metabolism

Creatine supplementation has been long used as means to drive muscle energy production and energetic capacity in athletes and bodybuilders and is the subject of a clinical trial as a potential therapeutic for UC (ClinicalTrials.gov Identifier: NCT02463305). ATP homeostasis is highly dependent on the efficient action of the creatine kinase (CK) circuit, as a large amount of cellular energy is stored as phosphocreatine. Creatine kinases utilize a large creatine pool (creatine + phosphocreatine) to distribute energy from regions of ATP production to regions of ATP consumption, acting as a temporal and, due to the subcellular compartmentalization of CKs, spatial ATP buffer. Creatine is equally provided physiologically by diet and endogenous production. Biosynthesis of creatine occurs simply through two enzymatic steps in which arginine and glycine are condensed by arginine:glycine aminotransferase (AGAT) to form guanidinoacetic acid (GAA) predominantly in the kidney, then methylation of GAA utilizing S-adenosyl methionine (SAM) by guanidinoacetate methyltransferase (GAMT) to form creatine in the liver, which is released into the bloodstream and taken up in other tissues.^136^ Inherent to this process is the transport of the zwitterionic GAA and creatine transport across cell membranes, which is mediated by two identified transporters – creatine transporter 1 (CrT) and monocarboxylate transporter 12 (MCT12). Tissues with high energy demand are abundant in CrT owing to its unidirectional transport and ability to concentrate intracellular molecules against a gradient, while MCT12 facilitates diffusion.^137^

Luminally sourced and endogenously synthesized creatine are both important for epithelial barrier function. Colonic epithelial cells localize CrT to their apical surfaces to utilize luminal substrate.^134^ At this time we are unaware of any studies characterizing a role for MCT12, which may contribute to IEC creatine metabolite exchange with the bloodstream. A murine diet fortified in creatine conferred enhanced AJC barrier resistance and decreased disease susceptibility in experimental models of colitis, with creatine kinases shown to localize to the AJC in support of ATP regeneration and to draw high energy phosphates to the region.^132^ This work also identified creatine kinases and CrT as HIF targets, further highlighting their importance to epithelial energy homeostasis and function. Moreover, mice deficient in endogenous creatine production due to AGAT mutation show increased AJC dysfunction and disease susceptibility to experimental colitis,^135^ while an inability to transport and utilize exogenous creatine is demonstrated to impede AJC formation and cells enriched in creatine transport show enhanced barrier formation (Table 1).^134^

Colonic creatine and creatinine, the spontaneous degradation product of phosphocreatine and creatine, are known to be utilized by the microbiota and may impact host physiology and pathology.^136^ Various bacteria are shown to express specific enzymes such as creatinine deaminase and creatine amidinohydrolase to facilitate creatinine and creatine break down.^133^ For example, several *Bacillus, Clostridia*, and *Escherichia* strains can degrade creatinine to 1-methylhydantion and ammonia for nitrogen procurement, while some *Pseudomonas, Brevibacterium*, and anaerobic *Clostridia* species can degrade the 1-methylhydantion further for nitrogen and carbon harvest.^136^ Additionally, GAA is degraded by several bacterial species such as *Corynebacterium* spp., *Pseudomonas aeruginosa*, and *Flavobacterium* spp., which are part of the normal human gut flora, through the enzyme guanidinoacetase.^138^ This bidirectional bacterial enzyme catalyzes the degradation of GAA with water to glycine and urea and vice versa. Increases in GAA were found in the gut of mice fed a high-fat diet and decreased in mice treated with metronidazole, suggesting a role for the microbiota in both the degradation and production of GAA.^139^ Altogether, a role for creatine metabolism in microbiota cross-feeding and microbiota–host energy circuits is apparent, but is clearly overlooked and incompletely understood.

## Disrupted microbiota–host energy circuits in ulcerative colitis

In 1980, Roediger reported that colonocytes obtained from UC patients were deficient in butyrate oxidation and postulated that the disease has a substantial “energy deficiency” component.^5^ In lieu of butyrate oxidation, the harvested colonocytes showed increased glucose and glutamine oxidation. An increase in glycolysis is a characteristic phenotype of proliferating cells to support the biomass accumulation necessary for cell division.^54^ In the normal large intestine, proliferating cells are confined to the lower two-thirds of the crypts. Indeed, patients with UC show more proliferating cells that extend high into the crypts, with extreme cases thought to be a state that precedes colon cancer.^140^ Rectal butyrate enemas in patients with active distal UC were found to reverse this proliferative phenotype and significantly reduce the number of proliferating cells in the upper 40% of crypts.^141^ Perhaps unsurprisingly, fecal butyrate levels are generally found diminished to varying degrees in UC,^142^ although it should be noted that fecal butyrate determinations represent a microbial butyrate production–host absorption balance and epithelial MCT1 (host butyrate absorption) is significantly diminished in UC.^143^ Such a decrease in host butyrate uptake likely results in an under-representation of the extent of fecal butyrate depletion, as decreased MCT1 itself, a target upregulated by butyrate,^144^ is indicative of decreased microbiota-derived butyrate. This lack of microbiota-derived butyrate coincides with diminished butyrate-producing species, notably *F. prausnitzii* and *Roseburia hominis*,^145^ and members of *Clostridium* cluster XIVa.^146^

Recent fecal microbiota transplantation (FMT) experiments assessing the efficacy of the treatment in UC and associated fecal microbiome and metabolome shifts provide invaluable insights into the compositional and metabolic dysbiosis of the disease state, and the changes from that state accompanying long-term remission, notably in lactate- and succinate-consuming energy circuits. In a healthy colon, lactate is typically found at less than 5 mM and succinate around 1–3 mM because of their function as microbial cross-feeding metabolites for butyrate and propionate formation, respectively.^89–91,98,142^ In UC, lactate can accumulate to 100 mM and succinate to 24 mM.^90,91^ Correspondingly, positive FMT outcomes associate with reinstation of the broken energy circuits contributing to diminished SCFA production and cross-feeding substrate accumulation. For instance, patients who maintain clinical remission show normalized levels of butyrate-producing and lactate-consuming members of *Clostridium* cluster IV (including *F. prausnitzii*) and XIVa (including *E. hallii* and *E. rectale*) with drastic concomitant reduction in fecal lactate levels.^146,147^ Furthermore, microbial taxa that discriminated positive therapy outcomes include the keystone species *R. bromii* and *A. muciniphilia* that degrade RS and mucin glycans, respectively, to provide mono/oligosaccharides and acetate for the aforementioned butyrate producers, with *A. muciniphilia* possibly contributing to the remission-associating decrease in fecal succinate.^147^ Of note, significant fecal metabolite increases in the salvageable purine nucleobases xanthine and adenine were observed upon FMT, indicating shifts in microbial purine metabolism, with sustained remission of UC correlating with decreases in fecal hypoxanthine and xanthine. As reinstatement of mucin production and restoration of the mucus barrier is inherent for the sustained remission of UC and concurrent habitat and fuel for *A. muciniphilia* and *Clostridium* cluster XIVa,^105^ and colonic epithelial utilization of microbiota-sourced purine is instrumental to mucin generation,^44^ it is intriguing to postulate a fundamental role for microbial purines in positive therapeutic outcomes.

## Future perspective: next-generation therapies

Interest in treating human diseases with beneficial microorganisms long predates recent discoveries, originating in a 1910 publication by Nobel laureate Elie Metchnikoff, *The Prolongation of Life*.^28^ Despite nearly three decades of effort to that end, probiotics have generally failed to live up to their expectations.^28^ Next-generation probiotics (NGPs) will include bacteria selected for a specific activity and genetically modified microorganisms (GMMs) designed for a specific function to consistently and locally deliver deficient microbiota-derived metabolites in diseases.^148^ The genetic engineering of probiotic strains offers therapeutic promise by endowing bacteria with beneficial functions to target a specific disease, and such GMMs have been made to treat diseases such as cancer, infections, metabolic disorders, and inflammation.^148,149^ Similar GMMs may be generated that are enriched in lactate and/or succinate consumption and SCFA and/or purine production to shift the IBD environment to a more homeostatic state and facilitate healing.

**Figure 3.**
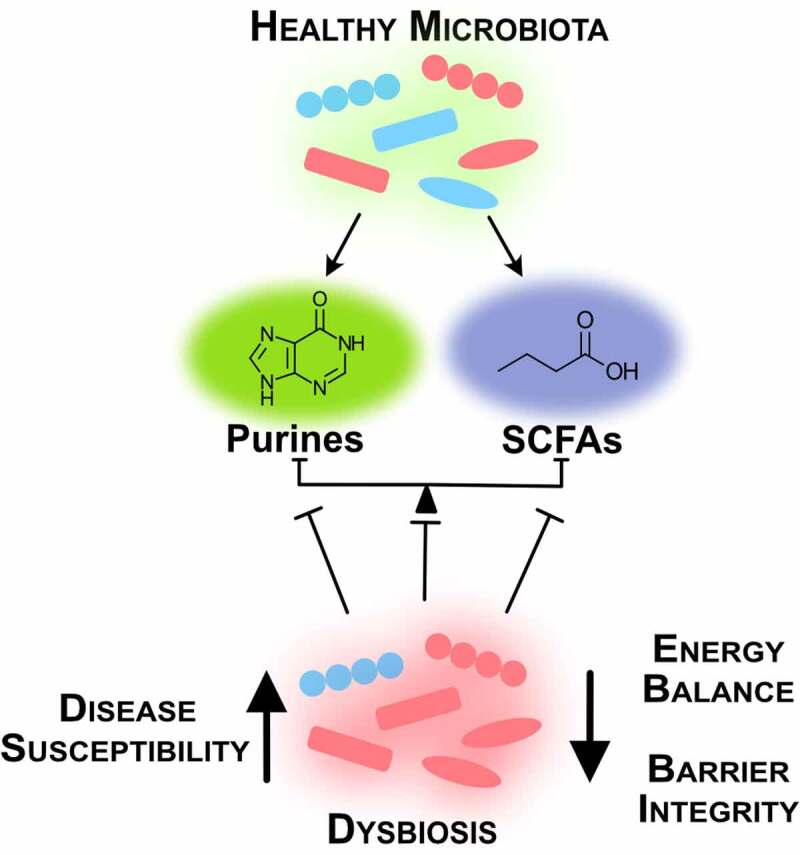
*Intestinal homeostasis requires balance between microbial species and their metabolites*. A healthy microbiota produces microbial metabolites crucial for intestinal function. Dysbiosis creates energy imbalance and loss of barrier function that lead to increased disease susceptibility (SCFAs, short-chain fatty acids)

A lesson derived from the FMT studies is that successful treatment of IBD is multifaceted and variable across individuals. Individually, microbial-derived metabolites such as butyrate and hypoxanthine exhibit specific influences on the gut epithelium, but as a diverse body of bacterial species and influential metabolites coexist in the human intestines, this plethora of microbes and their metabolic functions need to mesh in order to regulate energy metabolism and maintain homeostasis (Figure 3). Successful therapies may require assessment of diet, microbiota composition, microbiota and host metabolism, and epithelial barrier to understand what metabolic and functional circuits are broken and need reinstatement in each individual. From there, multifaceted treatments that address microbiota and host processes may involve bacteria selected for specific activities and/or GMMs, prebiotic supplementation (e.g. RS, oligosaccharides), small molecule supplementation (e.g. hypoxanthine, creatine), inflammatory suppression, and diet. Another lesson from FMTs is that sustained remission of UC appears possible through addressing dysbiosis of the composition and function of the microbiota, and processes required by the host mucosa for barrier function.

## Figures and Tables

**Table:** 

AJ	adherens junction
AJC	apical junction complex
AGAT	arginine:glycine aminotransferase
BMI	body mass index
CLDN1	Claudin 1
CR	Conventionally-raised
CK	creatine kinase
CrT1	creatine transporter 1
CD	Crohn’s disease
DSS	dextran sodium sulfate
ETC	electron transport chain
ER	endoplasmic reticulum
FMT	fecal microbiota transplantation
GI	Gastrointestinal
GMM	genetically modified microorganism
GF	germ-free
GAMT	guanidinoacetate methyltransferase
GAA	guanidinoacetic acid
HDAC	histone deacetylase
HIF	hypoxia-inducible factor
IEC	intestinal epithelial cell
IBD	inflammatory bowel disease
IBS	irritable bowel syndrome
MCT12	monocarboxylate transporter 12
MCT1	monocarboxylate transporter isoform 1
NGP	next-generation probiotic
NSP	non-starch polysaccharide
PRPP	phosphoribosyl pyrophosphate
RS	resistant starch
SAM	S-adenosyl methionine
SCFA	short chain fatty acid
SMCT1	sodium-coupled monocarboxylate transporter 1
TJ	tight junction
TAE	total available energy
UC	ulcerative colitis
UPR	unfolded protein response
XBP1	X-box binding protein 1
ZO	zona occludins
